# Adição da Troponina Ultrassensível à Avaliação de Risco Perioperatório Melhora a Capacidade Preditiva de Morte em Pacientes Submetidos à Cirurgia Não Cardíaca

**DOI:** 10.36660/abc.20230623

**Published:** 2024-04-17

**Authors:** Bruno Ferraz de Oliveira Gomes, Thiago Moreira Bastos da Silva, Giovanni Possamai Dutra, Leticia de Sousa Peres, Nathalia Duarte Camisão, Walter de Souza Homena, João Luiz Fernandes Petriz, Plinio Resende do Carmo, Basilio de Bragança Pereira, Gláucia Maria Moraes de Oliveira

**Affiliations:** 1 Hospital Barra D’Or Rio de Janeiro RJ Brasil Hospital Barra D’Or, Rio de Janeiro, RJ – Brasil; 2 Universidade Federal do Rio de Janeiro Instituto de Cardiologia Edson Saad Rio de Janeiro RJ Brasil Universidade Federal do Rio de Janeiro – Instituto de Cardiologia Edson Saad, Rio de Janeiro, RJ – Brasil

**Keywords:** Injúria Miocárdica, Cirurgia não Cardíaca, Troponina Ultrassensível, Classificação de Risco

## Abstract

**Fundamento:**

A estratificação ode risco é uma importante etapa na avaliação perioperatória. No entanto, os principais escores de risco não incorporam biomarcadores em seus conjuntos de variáveis.

**Objetivo:**

Avaliar o poder incremental da troponina à estratificação de risco tradicional.

**Métodos:**

Um total de 2230 pacientes admitidos na unidade de terapia intensiva após cirurgia não cardíaca foram classificados de acordo com três tipos de risco: Risco Cardiovascular (RCV), Índice de Risco Cardíaco Revisado (IRCR), e Risco Inerente da Cirurgia (RIC). O principal desfecho foi mortalidade por todas as causas. A regressão de Cox foi usada, assim como a estatística C antes e após a adição de troponina ultrassensível (pelo menos uma medida até três dias após a cirurgia). Finalmente, o índice de reclassificação líquida e a melhoria de discriminação integrada foram usadas para avaliar o poder incremental da troponina para a estratificação de risco. O nível de significância usado foi de 0,05.

**Resultados:**

A idade média dos pacientes foi 63,8 anos e 55,6% eram do sexo feminino. A prevalência de lesão miocárdica após cirurgia não cardíaca (MINS) foi 9,4%. Pacientes com um RCV elevado apresentaram uma maior ocorrência de MINS (40,1% x 24,8%, p<0,001), bem como pacientes com alto RIC (21,3 x 13,9%, p=0,004) e aqueles com IRCR≥3 (3,0 x 0,7%, p=0,009). Pacientes sem MINS, independentemente do risco avaliado, apresentaram taxa de mortalidade similar. A adição de troponina à avaliação de risco melhorou a capacidade preditiva de mortalidade em 30 dias e de mortalidade em um ano em todas as avaliações de risco.

**Conclusão:**

A prevalência de MINS é mais alta na população de alto risco. No entanto, sua prevalência na população de risco mais baixo não é desprezível e causa um maior risco de morte. A adição da troponina ultrassensível melhorou a capacidade preditiva da avaliação de risco em todos os grupos.

## Graphical abstract

**Figura Central:**
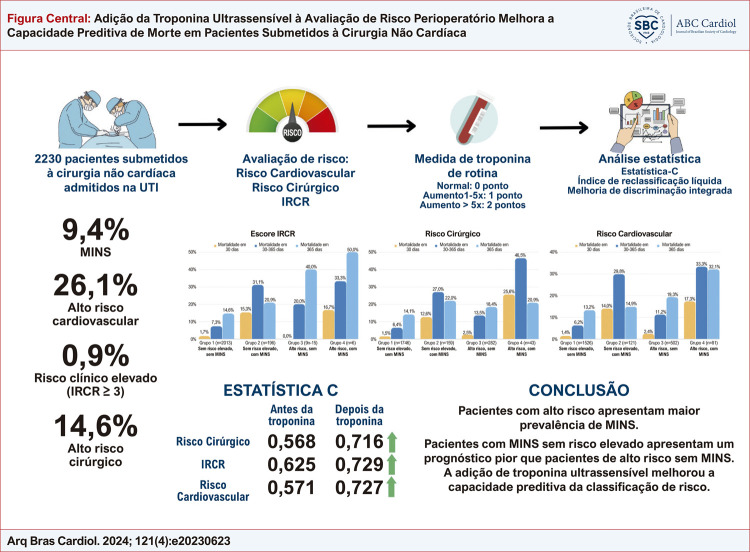
: Adição da Troponina Ultrassensível à Avaliação de Risco Perioperatório Melhora a Capacidade Preditiva de Morte em Pacientes Submetidos à Cirurgia Não Cardíaca

## Introdução

As complicações cardiovasculares são uma das principais causas de morte em pacientes submetidos a cirurgias não cardíaca em todo o mundo.^1,2^ Para minimizar e predizer essas complicações, sociedades internacionais de cardiologia e anestesiologia recomendam uma avaliação completa do risco cardiovascular antes da realização do procedimento proposto.^3^

As ferramentas disponíveis para a predição de risco são os escores de risco, os quais possuem capacidade preditiva limitada, principalmente quanto aos pacientes de risco mais baixo.^3,4^ A maioria dos escores de risco incorporam fatores de risco relacionados ao paciente e à cirurgia, mas não incluem biomarcadores em seus conjuntos de variáveis.^3^

A troponina ultrassensível é um marcador que denota lesão miocárdica, e sua elevação está relacionada a um risco aumentado de morte e eventos cardiovasculares em curto e em longo prazos.^5^ Apesar de sua boa capacidade preditiva, a troponina não foi incorporada aos principais escores de risco perioperatórios. Assim, são necessários novos estudos demonstrando seu valor incremental aos escores de risco existentes.

Uma vez que a lesão miocárdica ocorre em todos os estratos de risco, a troponina ultrassensível seria uma ferramenta potencial para a reclassificação de pacientes com baixo risco e que foram subdiagnosticados por meio de métodos tradicionais de avaliação de risco. Portanto, o objetivo deste estudo foi avaliar o comportamento da troponina ultrassensível em diferentes grupos de risco e o valor incremental desse biomarcador à estratificação usual de risco perioperatório em pacientes submetidos a cirurgias não cardíacas.

## Métodos

### População do estudo

Este estudo foi uma análise retrospectiva de dados prospectivos coletados do banco de dados local (isto é, amostra por conveniência). Foram incluídos pacientes submetidos à cirurgia não cardíaca e admitidos à Unidade de Terapia Intensiva (UTI). O período do estudo foi de janeiro de 2011 a dezembro de 2016. Os critérios de inclusão foram: permanência de pelo menos um dia UTI e presença de no mínimo uma dosagem de troponina ultrassensível em até três dias após a cirurgia. Pacientes submetidos a procedimentos cardíacos (por exemplo, cirurgia cardíaca, cateterismo, ablação, etc.) no último mês, que apresentaram estágios avançados da doença subjacente, e aqueles em cuidados paliativos foram excluídos do estudo.

Foram coletados dados de idade, sexo, fatores de risco clássicos (hipertensão, diabetes, doença coronária prévia, tabagismo, dislipidemia, insuficiência renal), tipo de cirurgia (geral, ortopédica, vascular, neurológica, torácica, de cabeça e pescoço, ginecológica e genitourinária), Índice de Risco Cardíaco Revisado (IRCR),^4^ risco intrínseco da cirurgia, e mensurações de troponina ultrassensível (pico e na admissão). Nesta UTI, a troponina ultrassensível é rotineiramente avaliada em todos os pacientes durante o período pós-operatório imediato e a partir do segundo dia de internação, exceto os pacientes com uma curta permanência na unidade. Pacientes que apresentaram níveis elevados de troponina apresentaram medidas seriadas até o valor mais alto (isto é, valor de pico).

A injúria miocárdica após cirurgia não cardíaca (MINS, do inglês *myocardial injury after noncardiac sugery*) foi definida como qualquer elevação nos níveis de troponina ultrassensível acima do ponto de corte (percentil 99) por até três dias após o procedimento cirúrgico, como recomendado pela *American Heart Association*.^6^ Para análise, consideraremos o valor mais alto de troponina observado nos três dias após a cirurgia. Durante o estudo, foram usados diferentes métodos de dosagem da troponina ultrassensível. Portanto, escolhemos avaliar a proporção de elevação de troponina de acordo com seu ponto de corte, fornecido pelo vendedor. O grau de elevação de troponina obtido pela razão entre o pico de troponina e o ponto de corte foi usado para criar três grupos: sem elevação de troponina, elevação até cinco vezes o ponto de corte, e elevação superior a cinco vezes o ponto de corte. A prevalência da lesão miocárdica foi avaliada em três grupos de risco: risco cardiovascular, risco clínico, e risco intrínseco à cirurgia.

Os critérios para determinar se um paciente tinha um alto risco cardiovascular foram: história de doença cardiovascular estabelecida (isto é, infarto do miocárdio prévio, acidente vascular cerebral ou doença arterial periférica), diabetes, doença renal crônica com *clearance* < 60mL/min, ou presença de pelo menos três fatores de risco (hipertensão, tabagismo, dislipidemia ou idade > 65 anos).

A definição de risco clínico elevado foi baseada em um IRCR ≥ 3, que indica um risco de morte, infarto, e parada cardiorrespiratória de aproximadamente 15% em 30 dias.^3^

Finalmente, a definição proposta pela diretriz da *European Society of Cardiology* foi usada para determinar se um paciente tinha um alto risco cirúrgico. Essa definição inclui vários procedimentos envolvendo risco de morte superior a 5%.^3^

A taxa de mortalidade foi avaliada consultando-se o banco de dados *online* de mortalidade do estado do Rio de Janeiro. O desfecho primário do estudo foi mortalidade por todas as causas e o tempo de acompanhamento mínimo no estudo foi de quatro anos. Nós avaliamos a ocorrência de morte em 30 dias, em um ano e após um ano.

### Análise estatística

A normalidade dos dados foi verificada usando o teste de Kolmogorov-Smirnov. As variáveis contínuas foram apresentadas em média e desvio padrão (quando há distribuição normal), ou mediana e intervalo interquartil (quando não há distribuição normal). As variáveis categóricas foram expressas em porcentagens. As variáveis foram comparadas de acordo com o desfecho primário usando a análise univariada com teste do qui-quadrado (variáveis categóricas) e teste de *Student* não pareado (variáveis contínuas).

Determinamos a prevalência da lesão miocárdica nos seguintes grupos de risco: pacientes com alto risco cardiovascular, pacientes com elevado risco clínico (IRCR ≥ 3) e pacientes com alto risco cirúrgico. Cada um desses grupos de risco foi avaliado em quatro subgrupos de acordo com a ocorrência ou não de lesão miocárdica – grupo 1: sem risco elevado com níveis normais de troponina; grupo 2: sem risco elevado com níveis elevados de troponina; grupo 3: com risco elevado e com níveis normais de troponina, e grupo 4: com risco elevado e com níveis elevados de troponina. Esses subgrupos foram avaliados usando a regressão de Cox ajustada pela gravidade (usando o escore SAPS3) e curvas de sobrevida para o desfecho primário. Cada um desses riscos foi avaliado por estatística C antes e após a adição de troponina de maneira categorizada (sem elevação de troponina; elevação de troponina 1-5x o ponto de corte; elevação de troponina > 5x o ponto de corte). Os escores para cada um desses itens corresponderam a números inteiros obtidos na regressão de Cox para os desfechos: morte em 30 dias e morte em um ano. O resultado da estatística C foi avaliado de acordo com a seguinte classificação: ruim (0,50 a <0,70), aceitável (0,70 a <0,80), excelente (0,80 a <0,90) e magnífico (≥0,90).^7^ Finalmente, o valor incremental de se adicionar troponina ao modelo de risco foi avaliado usando o Índice de Reclassificação Líquida (IRL)^8^ e o teste de melhoria de discriminação integrada (IDI, do inglês *integrated discrimination improvement*), com base nas categorias de risco.

Tanto o IRL como o IDI são medidas estatísticas usadas para avaliar o valor incremental de um novo teste diagnóstico ou prognóstico sobre um teste já existente. O IRL é uma medida da proporção de indivíduos que são corretamente reclassificados por um novo teste em comparação a um teste antigo. Ele é calculado como a diferença entre a proporção de indivíduos corretamente reclassificados para cima e a proporção dos indivíduos que são incorretamente reclassificados para baixo. O IDI é uma medida de melhoria na discriminação alcançada pelo novo teste em relação ao teste antigo. Ele é calculado como a diferença das probabilidades médias do novo teste e do teste antigo para indivíduos que apresentam um evento e as probabilidades médias para os indivíduos que não apresentam um evento. Ambos IRL e IDI são calculados utilizando modelos de regressão logística e podem ser usados para avaliar o valor incremental de um novo teste sobre um teste já existentes em termos de predição de risco.^8^

Para a análise estatística, foram usados os programas SPSS versão 26, MedCalc e RStudio 2021.09.0. Um p < 0,05 foi considerado estatisticamente significativo.

### Aspectos éticos

Este estudo foi registrado na Plataforma Brasil de acordo com o protocolo número CAAE 63829916.9.0000.5249 e aprovado pelo comitê de ética do Hospital Copa D’Or em 02 de fevereiro de 2017. Uma vez que este estudo foi uma análise retrospectiva, não foi necessário termo de consentimento.

## Resultados

### Características basais

Nós inicialmente identificados 2982 pacientes admitidos na UTI durante o período de estudo, mas, após analisar os critérios de inclusão, 2230 pacientes foram incluídos. Excluímos 495 pacientes por ausência de medidas de troponina, 35 por internações não cirúrgicas, 141 por procedimentos cardiovasculares, e 80 por tempo de internação inferior a 24 horas. Entre os pacientes excluídos que não possuíam medidas de troponina (a porcentagem mais alta de exclusão neste estudo, 80% permaneceram apenas um dia na UTI. Ocorreram sete óbitos nesse grupo, o que indica que esses pacientes apresentavam um perfil menos grave.

A prevalência de MINS foi de 9,4%. O tempo mediano de seguimento foi de 6,7 (IIQ 5,0-8,3) anos, com um tempo mediano de permanência na UTI de um dia, e um tempo mediano de permanência hospitalar de quatro dias. Um resumo dos resultados pode ser encontrado na Figura Central. As características gerais da população, bem como dos pacientes com e sem MINS são apresentadas na Tabela 1.

**Tabela 1:** – Características gerais da população com e sem injúria miocárdica após cirurgia não cardíaca

Características	Todos os pacientes N=2230	MINS N=209	Sem MINS N=2021	p
Idade média (anos)	63,8±16,3	73,2±13,4	60,7±15,8	<0,001
Sexo masculino	44,4%	45,9%	44,3%	0,350
IMC (Kg/m^2^)	29,7±20,0	27,9±23,2	30,2±18,8	0,04
Cirurgia de urgência	19,2%	33,0%	17,8%	<0,001
Insuficiência cardíaca prévia	1,8%	1,4%	1,8%	0,484
Doença renal crônica	4,1%	6,7%	3,9%	0,045
Hipertensão	62,8%	75,1%	61,5%	<0,001
Diabetes	25,7%	25,4%	25,8%	0,485
Doença coronariana prévia	7,6%	17,2%	6,6%	<0,001
Doença arterial periférica	2,4%	5,7%	2,0%	0,003
Fibrilação atrial	2,5%	5,3%	2,2%	0,013
Acidente vascular cerebral prévio	3,6%	3,3%	3,7%	0,504
Demência	4,0%	5,3%	3,9%	0,219
**Condição crônica de saúde**				
Independente	88,8%	76,1%	90,1%	<0,001
Necessidade de assistência	9,3%	19,1%	8,3%	
Restrito/acamado	1,9%	4,8%	1,6%	
Risco cardiovascular elevado	26,1%	40,1%	24,8%	<0,001
Risco clínico elevado (IRCR ≥ 3)	0,9%	3,0%	0,7%	0,009
Risco cirúrgico elevado (>5%)	14,6%	21,3%	13,9%	0,004
Óbito em longo prazo	24,9%	53,0 %	22,0%	<0,001

IMC: índice de massa corporal; IRCR: índice de risco cardíaco revisado.

Os principais fatores de risco cardiovascular identificados nesta população foram hipertensão arterial (62,8%) e diabetes (25,7%). Considerando todos as cirurgias realizadas, as mais comuns foram: geral (35%), ortopédica (36%), urológica (8,1%), vascular (5,2%) e neurológica (5,4%). Quase 15% das cirurgias foram consideradas de alto risco. Uma pequena proporção de pacientes de alto risco foi identificada pelo escore de risco IRCR (0,9%). Por outro lado, mais de um quarto dessa população preencheu os critérios para alto risco cardiovascular.

### Injúria miocárdica após cirurgia não cardíaca e mortalidade

Pacientes que apresentaram lesão miocárdica (grupos 2 e 4) mostraram taxas mais altas de mortalidade independentemente da classificação de risco empregada, principalmente no primeiro ano após a cirurgia.

A Figura S1 mostra a ocorrência de morte por todas as causas de acordo com o risco estimado e a ocorrência de lesão miocárdica. A Figura S2 mostra as taxas de mortalidade de acordo com o escore IRCR (disponível no material suplementar).

**Figura 1:**
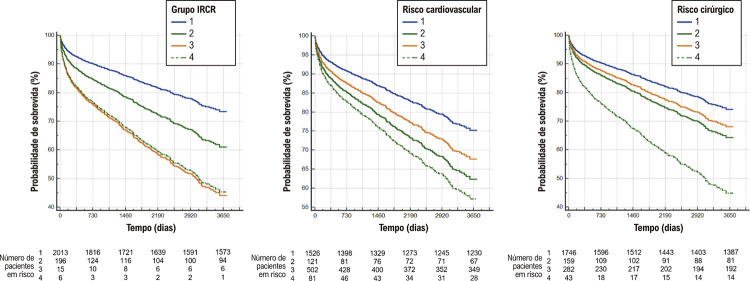
– Curvas de sobrevivência dos subgrupos estudados de acordo com o risco. Grupo 1: pacientes sem risco elevado sem injúria miocárdica; Grupo 2: pacientes sem risco elevado com injúria miocárdica; Grupo 3: pacientes com risco elevado sem injúria miocárdica; Grupo 4: pacientes com risco elevado e com injúria miocárdica. IRCR: índice de risco cardíaco revisado.

A Figura 1 mostra as curvas de sobrevida dos grupos segundo a classificação de risco utilizada.

Em todas as curvas de sobrevida, exceto aquela determinada pelo IRCR, observamos taxas mais altas de mortalidade nos grupos com lesão miocárdica. Quando utilizamos o escore IRCR para definir o risco, pacientes de alto risco apresentaram taxas de mortalidade mais altas.

Na Tabela 2, apresentamos a regressão de Cox ajustada pelo escore SAPS3 (como uma variável contínua) e respectivos valores de razão de risco (*hazard ratios*) para mortalidade em longo prazo segundo a análise de risco usada.

**Tabela 2:** – Regressão de Cox ajustada por gravidade utilizando o escore SAPS3 para o desfecho de mortalidade em longo prazo

	Risco cirúrgico		Risco clínico (IRCR)		Risco cardiovascular	
			
	HR	IC95%	HR	IC95%	HR	IC95%
**Grupo 1**	***referência***		***referência***		***referência***	
**Grupo 2**	1,48	1,12-1,94	1,60	1,26-2,03	1,65	1,22-2,24
**Grupo 3**	1,29	1,02-1,63	2,64	1,36-5,13	1,37	1,23-1,67
**Grupo 4**	2,68	1,81-3,95	2,55	1,05-6,20	1,96	1,42-2,71
**SAPS3**	1,06	1,05-1,07	1,06	1,05-1,07	1,06	1,05-1,07

IRCR: índice de risco cardíaco revisado; HR: hazard ratio; IC95%: intervalo de confiança de 95%

Conforme observado nas curvas de sobrevida, a lesão miocárdica levou a uma maior mortalidade independentemente do risco, exceto na população estratificada pelo IRCR.

### Troponina e estratificação de risco

**Figura 2:**
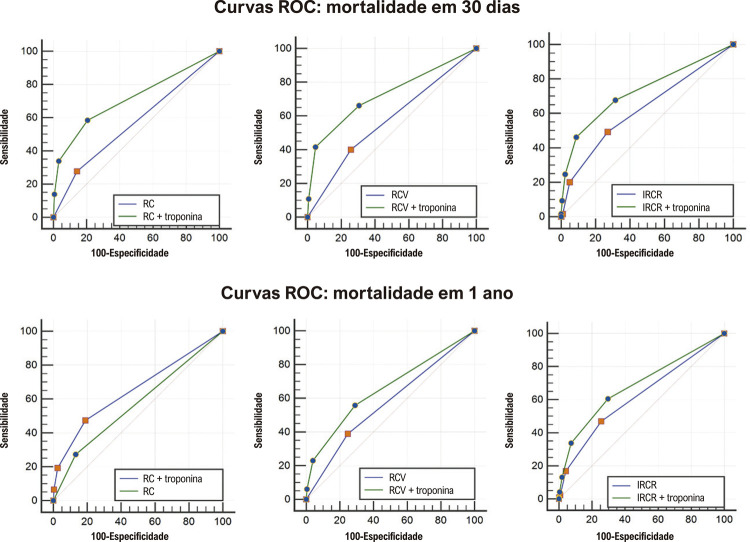
– Curva ROC para cada risco antes e após a adição da troponina para os desfechos: mortalidade em 30 dias e mortalidade em um ano. RC: risco cirúrgico; RCV: risco cardiovascular; IRCR: índice de risco cardíaco revisado.

Como a troponina teve um impacto maior sobre a mortalidade até um ano após o procedimento cirúrgico, escolhemos analisar o poder incremental, dados os escores de risco em 30 dias e em um ano. A regressão de Cox foi usada para determinar os coeficientes para a adição de troponina, que estão disponíveis no material suplementar (Tabela S1). Essa regressão determinou que um aumento de um a cinco vezes nos níveis de troponina adicionaria um ponto, e um aumento superior a cinco vezes o ponto de corte da troponina adicionaria dois pontos ao escore usado –risco cirúrgico elevado = 1 ponto, risco cardiovascular elevado = 1 ponto, e escore IRCR (0-6 pontos). Os escores foram avaliados antes e após a incorporação da troponina usando a curva ROC e a estatística C. Os resultados são mostrados na Figura 2 e na Tabela 3.

**Tabela 3:** – Estatística C antes e após a adição de troponina para os desfechos: mortalidade em 30 dias e mortalidade em um ano

	RC x RC + troponina	IRCR x IRCR + troponina	RCV x RCV + troponina
Mortalidade em 30 dias	0,568 x 0,716*	0,625 x 0,729*	0,571 x 0,727*
Mortalidade em um ano	0,570 x 0,655*	0,618 x 0,684*	0,571 x 0,657*

RC: risco cirúrgico; IRCR: índice de risco cardíaco revisado; RCV: risco cardiovascular; *p < 0,001.

**Figura 3:**
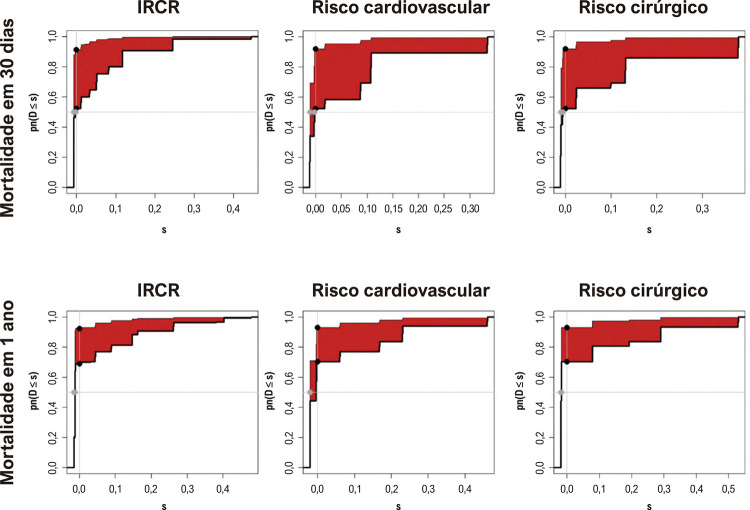
– Representação gráfica da melhoria de discriminação integrada para cada escore em dois desfechos diferentes (mortalidade em 30 dias e mortalidade em um ano). A área sob a curva do gráfico é uma medida de melhoria (área vermelha) na discriminação alcançada pelo teste novo em relação ao teste antigo; uma maior área sob a curva indica maior melhoria na discriminação; p<0,001 em todas as curvas, valores numéricos são disponíveis no material suplementar (Tabela 2) .

Em todos os grupos de risco e desfechos, a adição da troponina aumentou significativamente a acurácia do escore de risco. Para o desfecho mortalidade em 30 dias e mortalidade em um ano, todos os escores de risco apresentaram baixa acurácia. O escore com a acurácia mais alta foi o IRCR, tanto para a taxa de mortalidade em 30 dias como para a taxa de mortalidade em um ano. Por outro lado, após a adição de troponina, todos os escores de risco mostraram uma acurácia similar, mas ainda aceitável ou mesmo baixa, principalmente na avaliação da mortalidade em um ano. Na análise do valor incremental (Tabela 4 e Figura 3), observamos um valor incremental em todos os modelos de risco estudados, especialmente na mortalidade em 30 dias.

**Tabela 4:** – Adição da troponina aos escores de risco usando o Índice de Reclassificação Líquida (IRL)

	RC x RC + troponina			IRCR x IRCR + troponina			RCV x RCV + troponina		
			
	IRLe	IRLae	IRL	IRLe	IRLae	IRL	IRLe	IRLae	IRL
Mortalidade em 30 dias	0,48	0,08	0,40	0,48	0,08	0,40	0,48	0,08	0,40
Mortalidade em um ano	0,30	0,07	0,23	0,30	0,07	0,23	0,30	0,07	0,23

RC: risco cirúrgico; IRCR: índice de risco cardíaco revisado; RCV: risco cardiovascular; IRLe: IRL de eventos; IRLae: IRL de ausência de eventos.

## Discussão

Neste estudo, observamos uma maior prevalência de injúria miocárdica em pacientes com risco mais alto, incluindo risco cardiovascular, cirúrgico e clínico. No entanto, a ocorrência de MINS na população sem alto risco não é desprezível e causa alta mortalidade nessa população. No acompanhamento em longo prazo, os pacientes sem risco elevado e injúria miocárdica apresentaram pior prognóstico que aqueles com risco elevado sem injúria miocárdica. A avaliação tradicional de risco mostrou baixa acurácia na predição de morte em 30 dias e em um ano. Em contrapartida, a adição da investigação de injúria miocárdica através da troponina ultrassensível permitiu aumentar a acurácia da predição desses eventos, especialmente na população sem alto risco.

Sabe-se que a MINS aumenta o risco de morte em curto prazo e em longo prazo,^5^ mas o papel da avaliação de risco nesse aumento foi pouco estudado. Analisando os fatores de risco individualmente, sabemos que a hipertensão, o diabetes e o tabagismo aumentam o risco de MINS.^9^ Entretanto, não temos estudos avaliando o paciente de alto risco cardiovascular diante da ocorrência de MINS. Na avaliação inicial do risco pré-operatório do paciente, as diretrizes recomendam a avaliação dos fatores de risco e da presença de doença cardiovascular estabelecida.^3^ Para um paciente com idade igual ou superior a 65 anos com fatores de risco ou doença cardiovascular estabelecida, recomenda-se realizar um eletrocardiograma e medir biomarcadores (troponina e BNP). Porém, esses dados não são usados nos escores de risco.

Em nosso estudo, a prevalência de alto risco cardiovascular foi considerável, assim como a prevalência de hipertensão (62,8%) e diabetes (25,7%). Pacientes com alto risco cardiovascular apresentaram uma maior prevalência de MINS (40,1 x 24,8%). Por outro lado, a taxa de mortalidade de 30 dias de pacientes com alto risco cardiovascular sem MINS foi similar à dos pacientes sem risco cardiovascular elevado (2,4 x 1,4%). Assim, observamos que quase um quarto da população sem risco cardiovascular elevado apresentou MINS, o que determinou uma mortalidade em 30 dias mais alta (14%) e em um ano (29,8%), e demonstrou a necessidade de um rastreamento para MINS mesmo em pacientes sem um risco cardiovascular elevado. Mesmo após ajuste quanto à gravidade, esses pacientes apresentaram um pior prognóstico em um seguimento de quase sete anos.

Quando avaliamos pacientes submetidos à cirurgia de alto risco, encontramos resultados semelhantes. Embora alguns escores de risco incluam o risco intrínseco da cirurgia,^3^ há poucos dados disponíveis acerca da ocorrência de MINS e seu impacto prognóstico. Em nossa população, a prevalência de cirurgia de alto risco foi de 14,6%, e esses pacientes apresentaram uma prevalência mais alta de MINS (21,3% vs. 13,9%). Na ausência de MINS, a taxa de mortalidade em 30 dias desses pacientes foi similar à de pacientes submetidos a cirurgias de risco mais baixo (2,5% vs. 1,5%). No entanto, a ocorrência de MINS aumenta o risco de morte independentemente da cirurgia realizada, com uma taxa de mortalidade em 30 dias de 12,6% em pacientes submetidos à cirurgia de risco mais baixo. Esse achado foi consistente ao longo do estudo, indicando que a avaliação do risco exclusivamente pela análise do risco inerente à cirurgia é inadequada.

Por fim, nós analisamos pacientes em alto risco usando o IRCR, um dos escores de risco pré-operatório mais utilizados na prática clínica. Neste estudo, somente 0,9% foram considerados de alto risco (IRCR ≥ 3). Mesmo assim, esses pacientes apresentaram uma maior prevalência de MINS (3,0 x 0,7%). Na análise de mortalidade em 30 dias, não observamos ocorrência de óbito entre os pacientes com alto IRCR e sem MINS. No entanto, a ocorrência de MINS foi associada com 16,7% das mortes nesse grupo. Na análise da mortalidade em longo prazo, observamos que os grupos com alto IRCR (com e sem MINS) apresentaram um pior prognóstico no seguimento. Esse achado pode ser justificado pelo pequeno tamanho amostral do grupo (64 pacientes), em que a ocorrência de um evento foi exacerbada em relação ao outro grupo.

O IRCR, embora amplamente utilizado, não é uma ferramenta com boa acurácia em detectar eventos cardiovasculares, principalmente na mortalidade por todas as causas.^10,11^ A acurácia detectada em nosso estudo (estatística C = 0,625 para mortalidade em 30 dias) está de acordo com a encontrada na literatura.^3^ No entanto, a adição de troponina ultrassensível obtida no pós-operatório conseguiu aumentar sua capacidade preditiva. Vasireddi et al.^12^demonstraram que pacientes classificados como de baixo risco pelo escore IRCR apresentaram taxas mais altas de mortalidade quando apresentavam lesão miocárdica,^12^ um achado corroborado por este estudo. Pelo fato de pacientes de baixo risco serem geralmente negligenciados quanto à tomada de medidas protetivas no pré-operatório, eles poderiam ser mais expostos ao risco de lesão miocárdica. Esse achado foi consistente com outras avaliações de risco, como a do risco inerente da cirurgia e do risco cardiovascular. Apesar do aumento na capacidade preditiva com a adição de troponina ultrassensível à estratificação de risco, a acurácia foi considerada somente aceitável (estatística C entre 0,7 e 0,8).^13,14^ Assim, novos escores de avaliação de risco incluindo troponina ultrassensível ainda não necessários.

Na análise utilizando o IRL, observamos uma maior taxa de reclassificação em pacientes que apresentaram lesão miocárdica, principalmente quanto à mortalidade em 30 dias. Esse achado corrobora o poder incremental da troponina ultrassensível na reclassificação de risco de pacientes submetidos a cirurgias não cardíacas. Nossos resultados foram ainda reforçados pelos resultados do teste IDI. O IDI é uma ferramenta amplamente utilizada para avaliar a capacidade de um marcador em predizer desfechos binários. Tem se sugerido que o IDI seja mais sensível que outras métricas na identificação de preditores úteis de mortalidade em pacientes submetidos à cirurgia não cardíaca. Isso foi demonstrada utilizando-se três métodos estatísticos distintos, adicionando robustez aos nossos resultados.

A adição da troponina ultrassensível à prática clínica permitiu a detecção de pequenos graus de lesão miocárdica. O seguimento com troponina ultrassensível do estudo VISION demonstrou que elevações acima de 5ng/L no pós-operatório de cirurgias não cardíacas aumentam a mortalidade em 30 dias.^15^ Em nosso estudo, demonstramos que pacientes em baixo risco também são vulneráveis à lesão miocárdica. Por outro lado, a população estudada tinha um risco potencial de doença grave, considerando seu tempo de internação na UTI superior a uma noite. Assim, essa população merece um rastreamento de rotina com dosagem de troponina ultrassensível no período pós-operatório, independentemente do risco, um achado também corroborado por nosso estudo.

O presente estudo apresenta algumas limitações. Apesar de ser um estudo de análise retrospectiva, os dados foram prospectivamente coletados do banco de dados local. Diferentes kits de troponina foram usados durante o estudo, dificultando a padronização dos dados como uma variável contínua. De qualquer maneira, a recomendação da *American Heart Association* é, independentemente do kit utilizado, usar o percentil 99 para caracterizar os pacientes com MINS. Além dessas limitações, este é um estudo unicêntrico. Ainda, quando analisamos os desfechos de longo prazo, outros fatores podem diretamente influenciar o risco de morte, que não podem ser controlados em um estudo retrospectivo, adicionando um alto risco de viés. Finalmente, a seleção de pacientes admitidos na UTI demonstra uma população potencialmente de maior risco e, portanto, nossos resultados não podem ser extrapolados para outras populações.

Apesar dessas limitações, nosso estudo está entre os poucos que avaliaram o prognóstico de pacientes de riscos muito diferentes, submetidos a cirurgias não cardíacas. Nós empregamos três classificações de risco e demonstramos que mesmo pacientes considerados de baixo risco podem ser expostos a uma mortalidade mais alta. Nossos resultados destacam a necessidade de um uso mais amplo de medidas de troponina ultrassensível na identificação de pacientes em maior risco. Escores e avaliações de risco atuais falham em detectar esses pacientes, a adição de troponina ultrassensível melhorou a capacidade preditiva para mortalidade em 30 dias e mortalidade em um ano.

## Conclusões

Pacientes considerados em alto risco com base no risco cardiovascular, risco inerente da cirurgia ou escore IRCR apresentaram uma prevalência mais alta de injúria miocárdica quando submetidos à cirurgia não cardíaca. A estratificação de risco convencional mostrou baixa acurácia em predizer mortalidade por todas as causas em curto e em longo prazo. A adição da troponina ultrassensível para a avaliação de risco aumentou sua capacidade preditiva, mas ainda é insuficiente para uma boa predição de eventos. Novos escores usando biomarcadores devem ser desenvolvidos.
