# Supplementary material for: Adição da Troponina Ultrassensível à Avaliação de Risco Perioperatório Melhora a Capacidade Preditiva de Morte em Pacientes Submetidos à Cirurgia Não Cardíaca
Source: Arq Bras Cardiol. 2024 Apr 17;121(4):e20230623. [Article in Portuguese] doi: 10.36660/abc.20230623 (PMC11098574; doi:10.36660/abc.20230623)
Supplement: Translation

# Translation: Addition of High-Sensitivity Troponin to Perioperative Risk Assessment Improves the Predictive Ability of Death in Non-Cardiac Surgery Patients

**DOI:** 10.36660/abc.20230623i

**Authors:** Bruno Ferraz de Oliveira Gomes, Thiago Moreira Bastos da Silva, Giovanni Possamai Dutra, Leticia de Sousa Peres, Nathalia Duarte Camisão, Walter de Souza Homena, João Luiz Fernandes Petriz, Plinio Resende do Carmo, Basilio de Bragança Pereira, Gláucia Maria Moraes de Oliveira

**Affiliations:** 1 Brazil Hospital Barra D’Or, Rio de Janeiro, RJ – Brazil; 2 Brazil Universidade Federal do Rio de Janeiro – Instituto de Cardiologia Edson Saad, Rio de Janeiro, RJ – Brazil

**Keywords:** Myocardial Injury, Non-cardiac Surgery, High-sensitivity Troponin, Risk Classification

## Abstract

**Background:**

Risk stratification is an important step in perioperative evaluation. However, the main risk scores do not incorporate biomarkers in their set of variables.

**Objective:**

Evaluate the incremental power of troponin to the usual risk stratification

**Methods:**

A total of 2,230 patients admitted to the intensive care unit after non-cardiac surgery were classified according to three types of risk: cardiovascular risk (CVR), Revised Cardiac Risk Index (RCRI); and inherent risk of surgery (IRS). The main outcome was all-cause mortality. Cox regression was used as well as c-statistics before and after addition of high-sensitivity troponin (at least one measurement up to three days after surgery). Finally, net reclassification index and integrated discrimination improvement were used to assess the incremental power of troponin for risk stratification. Significance level was set at 0.05.

**Results:**

Mean age of patients was 63.8 years and 55.6% were women. The prevalence of myocardial injury after non-cardiac surgery (MINS) was 9.4%. High CVR-patients had a higher occurrence of MINS (40.1 x 24.8%, p<0.001), as well as high IRS-patients (21.3 x 13.9%, p=0.004) and those with a RCRI≥3 (3.0 x 0.7%, p=0.009). Patients without MINS, regardless of the assessed risk, had similar mortality rate. The addition of troponin to the risk assessment improved the predictive ability of death at 30 days and at 1 year in all risk assessments.

**Conclusion:**

The prevalence of MINS is higher in the high-risk population. However, its prevalence in lower-risk population is not negligible and causes a higher risk of death. The addition of high-sensitivity troponin increased the predictive ability of risk assessment in all groups.

## Introduction

Cardiovascular complications are one of the main causes of death in patients undergoing non-cardiac surgeries worldwide.^1,2^ In order to minimize and predict these complications, international societies of cardiology and anesthesiology recommend a thorough assessment of cardiovascular risk before performing the proposed procedure.^3^

The tools available for risk prediction are risk scores, which have limited predictive capacity, especially regarding patients at lower risk.^3,4^ Most risk scores incorporate patient and surgery-related risk factors, but do not include biomarkers in their set of variables.^3^

High-sensitivity troponin is a biomarker that denotes myocardial injury, and its elevation is related to an increased risk of death and cardiovascular events in the short and long terms.^5^ Despite its good predictive capacity, troponin has not been incorporated into the main perioperative risk scores. Thus, new studies demonstrating its incremental value to the existing risk scores are needed.

As myocardial injury occurs in all risk strata, high-sensitivity troponin would be a potential tool for risk reclassification of low-risk patients who were underdiagnosed by traditional assessment methods. Therefore, the objective of this study is to evaluate the behavior of high-sensitivity troponin in different risk groups and the incremental value of this biomarker to the usual perioperative risk stratification in patients undergoing non-cardiac surgeries.

## Methods

### Study Population

This is a retrospective analysis study using prospective data collected from the local database (i.e., convenience sample). Patients who underwent non-cardiac surgery and were admitted to an intensive care unit (ICU) were included. The study period was from January 2011 to December 2016. The inclusion criteria were the following: at least an overnight stay in ICU and a minimum of one high-sensitivity troponin dosage up to three days after surgery. Patients who underwent cardiac procedures (e.g., cardiac surgery, catheterization, ablation, etc.) in the last month, presented advanced stage of the underlying disease, and those on palliative care were excluded from the study.

Data on age, gender, classic risk factors (hypertension, diabetes, previous coronary disease, smoking, dyslipidemia, renal failure), type of surgery (general, orthopedic, vascular, neurological, chest, head and neck, and gynecological and genitourinary), revised cardiac risk index (RCRI)^4^ risk score, surgery risk assessment, admission and peak high-sensitivity troponin levels were collected. In this ICU, high-sensitivity troponin is routinely checked in all patients during the immediate post-operative period and from the second day of hospitalization, except patients with a short stay in the unit. Patients who showed elevated troponin levels had serial measurements up to the highest value (i.e., peak troponin).

Myocardial injury after non-cardiac surgery (MINS) was defined as any elevation of high-sensitivity troponin above the cut-off point (99th percentile) for up to three days after the surgical procedure, as recommended by the American Heart Association.^6^ For analysis, we will consider the highest value of troponin in the three post-operative days. During the study, different high-sensitivity troponin assays were used. Therefore, we chose to evaluate the proportion of troponin elevation according to its cutoff point, provided by the vendor. The degree of troponin elevation obtained through the ratio between troponin peak and cut-off point was used to create three groups, namely: no troponin elevation, elevation up to five times the cut-off point, and elevation greater than five times the cut-off point. The prevalence of myocardial injury was evaluated in three risk groups as follows: cardiovascular risk, clinical risk, and intrinsic risk of surgery.

The criteria for determining whether a patient was at high cardiovascular risk were the following: history of established cardiovascular disease (i.e., previous myocardial infarction, stroke or peripheral arterial disease), diabetes, chronic kidney disease with clearance < 60ml/min, or presence of at least three risk factors (i.e., hypertension, smoking, dyslipidemia or age > 65 years).

The definition of high clinical risk was based on a RCRI score ≥ 3, which indicates a risk of death, infarction, or cardiorespiratory arrest of approximately 15% within 30 days.^3^

Finally, the definition proposed by the European Society of Cardiology guideline was used to determine whether a patient was at a high surgical risk. It includes several procedures involving risk of death greater than 5%.^3^

Mortality rate was assessed by consulting the online mortality database of the state of Rio de Janeiro. The primary outcome of this study was all-cause mortality and the minimum follow-up time in the study was four years. We evaluated the occurrence of death at 30 days, at one year and one year thereafter.

### Statistical analysis

Data normality was verified by using the Kolmogorov-Smirnov test. Continuous variables were presented as mean and standard deviation (when there is normal distribution) or median and interquartile range (when there is no normal distribution). Categorical variables were expressed as percentages. The variables were compared according to the primary outcome by using univariate analysis with chi-square test (categorical variables) and unpaired Student’s t test (continuous variables).

We determined the prevalence of myocardial injury in the following risk groups: patients at high cardiovascular risk, high clinical risk (RCRI ≥ 3) and patients at high surgical risk. Each of these risk groups were evaluated in four subgroups according to the occurrence or not of myocardial injury – group 1: non-high-risk with normal troponin levels; group 2: non-high-risk with elevated troponin levels; group 3: high-risk with normal troponin levels, and group 4: high- risk with elevated troponin levels. These subgroups were evaluated by using Cox regression adjusted for severity (using SAPS3 score) and survival curves for primary outcome. Each of these risks was assessed by using c-statistics before and after adding troponin in a categorized manner (no troponin elevation; troponin elevation 1-5x the cutoff point; troponin elevation > 5x cutoff point). The scores for each of these items corresponded to the integer values obtained in the Cox regression for the outcomes: death at 30 days and death at one year. The c-statistic result was assessed according to the following classification: poor (0.50 to <0.70), acceptable (0.70 to <0.80), excellent (0.80 to <0.90) and magnificent (≥0.90).^7^ Finally, the incremental value of adding troponin to the risk model was evaluated by using the net reclassification index (NRI)^8^and integrated discrimination improvement (IDI) test based on the risk categories.

Both NRI and IDI are statistical measures that are used to evaluate the incremental value of a new diagnostic or prognostic test over an existing one. The NRI is a measure of the proportion of individuals who are correctly reclassified by the new test compared to the old test. It is calculated as the difference between the proportion of individuals who are correctly reclassified upwards and the proportion of individuals who are incorrectly reclassified downwards. The IDI is a measure of the improvement in discrimination that is achieved by the new test over the old test. It is calculated as the difference between the mean predicted probabilities of the new test and the old test for individuals who experience an event minus the mean predicted probabilities for individuals who do not experience an event. Both the NRI and IDI are calculated using logistic regression models and can be used to evaluate the incremental value of a new test over an existing one in terms of risk prediction.^8^

For statistical analysis, the SPSS software version 26, MedCalc and RStudio 2021.09.0 software were used. P < 0.05 was considered statistically significant.

### Ethical aspects

This study was registered on Plataforma Brasil (protocol number CAAE 63829916.9.0000.5249) and approved by the research ethics committee of the Copa D’Or Hospital on February 2, 2017. Because it is a retrospective analysis study, no informed consent form was required.

## Results

### Baseline characteristics

We initially identified 2,982 patients admitted to ICU during the study period, but after analyzing the inclusion criteria, 2,230 patients were included. We excluded 495 patients due to lack of troponin measurement, 35 due to non-surgical hospitalizations, 141 due to cardiovascular procedures, and 80 due to hospitalization less than 24 hours. Among the excluded patients who had no troponin measurement (the highest percentage of exclusion in this study), 80% stayed only one day in the ICU. There were seven deaths in this group, which indicates these patients had a less severe profile.

The prevalence of MINS was 9.4%. The median follow-up time was 6.7 (IQR 5.0-8.3) years, with a median ICU stay of one day and a median hospital stay of four days. A summary of the results can be found in the Central Illustration. General characteristics of the population, as well as of the patients with and without MINS, are shown in Table 1.

**Table 1:** – General characteristics of the population and patients with myocardial injury after noncardiac surgery

Caracteristics	All patients N=2230	MINS N=209	Non-MINS N=2021	p
Mean age (years)	63.8±16.3	73.2±13.4	60.7±15.8	<0.001
Male gender	44.4%	45.9%	44.3%	0.350
BMI (Kg/m^2^)	29.7±20.0	27.9±23.2	30.2±18.8	0.04
Urgent surgery	19.2%	33.0%	17.8%	<0.001
Previous heart failure	1.8%	1.4%	1.8%	0.484
Chronic kidney disease	4.1%	6.7%	3.9%	0.045
Hypertension	62.8%	75.1%	61.5%	<0.001
Diabetes	25.7%	25.4%	25.8%	0.485
Previous myocardial infarction	7.6%	17.2%	6.6%	<0.001
Peripheral artery disease	2.4%	5.7%	2.0%	0.003
Atrial fibrillation	2.5%	5.3%	2.2%	0.013
Previous stroke	3.6%	3.3%	3.7%	0.504
Dementia	4.0%	5.3%	3.9%	0.219
**Chronic health status**				
Independence	88.8%	76.1%	90.1%	<0.001
Need for assistance	9.3%	19.1%	8.3%	
Restricted/bedridden	1.9%	4.8%	1.6%	
High cardiovascular risk	26.1%	40.1%	24.8%	<0.001
High clinical risk (RCRI ≥ 3)	0.9%	3.0%	0.7%	0.009
High surgical risk (>5%)	14.6%	21.3%	13.9%	0.004
Long-term death	24.9%	53.0 %	22.0%	<0.001

BMI: body mass index; RCRI: revised cardiac risk index.

The main cardiovascular risk factors identified in this population were arterial hypertension (62.8%) and diabetes (25.7%). Considering all surgeries performed, the most common were general (35%), orthopedic (36%), urological (8.1%), vascular (5.2%) and neurological (5.4%). Nearly 15% of the surgeries were considered of high risk. A low proportion of high-risk patients was identified by the RCRI score (0.9%). In contrast, more than a quarter of this population met the criteria for high cardiovascular risk.

### MINS and mortality

Patients who presented myocardial injury (Groups 2 and 4) showed higher mortality rates regardless of the risk classification used, especially in the first year after surgery.

Figure S1 shows the occurrence of all-cause death according to the estimated risk and occurrence of myocardial injury and Figure S2 shows mortality rates according to the RCRI score (available in supplementary material).

Figure 1 shows the survival curves of the groups according to the risk classification used.

**Figure 1:** – Survival curves of the studied subgroups according to risk. Group 1: non-high-risk patients without myocardial injury; Group 2: non-high-risk patients with myocardial injury; Group 3: high-risk patients without myocardial injury; Group 4: high-risk patients with myocardial injury. RCRI: revised cardiac risk index.

In all survival curves, except the one determined by the RCRI, we observed higher mortality rates in the groups with myocardial injury. When we used the RCRI score to define risk, high-risk patients had higher mortality rates.

In Table 2, we provide the Cox regression adjusted for SAPS3 score (as a continuous variable) and their respective hazard ratios for long-term death according to the risk analysis used.

**Table 2:** – Cox regression adjusted for severity by using the SAPS3 score for the outcome long-term death

	Surgical Risk		Clinical Risk (RCRI)		Cardiovascular risk	
			
	HR	95%CI	HR	95%CI	HR	95%CI
**Group 1**	***reference***		***reference***		***reference***	
**Group 2**	1.48	1.12-1.94	1.60	1.26-2.03	1.65	1.22-2.24
**Group 3**	1.29	1.02-1.63	2.64	1.36-5.13	1.37	1.23-1.67
**Group 4**	2.68	1.81-3.95	2.55	1.05-6.20	1.96	1.42-2.71
**SAPS3**	1.06	1.05-1.07	1.06	1.05-1.07	1.06	1.05-1.07

RCRI: revised cardiac risk index; HR: hazard ratio; 95%CI: 95% confidence interval.

As observed in the survival curves, myocardial injury led greater mortality regardless of the risk, except in the population stratified by RCRI.

### Troponin and risk stratification

As troponin had a greater impact on mortality up to one year after the surgical procedure, we chose to analyze the incremental power given the risk scores at 30 days and at one year. Cox regression was used to determine the coefficients for the addition of troponin, which are available in the supplementary material (Table S1). This regression determined that 1-5 times increase in troponin levels would add one point, and an increase greater than five times the troponin cutoff point would add two points to the score used, namely: high surgical risk = 1 point, high cardiovascular risk = 1 point, and RCRI score (0-6 points). Scores were evaluated before and after troponin incorporation by using ROC curve and c-statistical analysis. The results are shown in Figure 2 and Table 3.

**Figure 2:** – ROC curve for each of the risks before and after addition of troponin for the outcomes mortality at 30 days and mortality at one year; SR: surgical risk; CVR: cardiovascular risk; RCRI: revised cardiac risk index.

**Table 3:** – C-statistics before and after addition of troponin for the outcomes mortality at 30 days and mortality at one year

	SR x SR + troponin	RCRI x RCRI + troponin	CVR x CVR + troponin
30-day mortality	0.568 x 0.716*	0.625 x 0.729*	0.571 x 0.727*
One-year mortality	0.570 x 0.655*	0.618 x 0.684*	0.571 x 0.657*

SR: surgical risk; RCRI: revised cardiac risk index; CVR: cardiovascular risk. *p < 0.001.

In all risk groups and outcomes, the addition of troponin significantly increased the accuracy of the risk score. For the outcomes mortality at 30 days and mortality at one year, all risk scores had poor accuracy. The score with the highest accuracy was the RCRI, both for 30-day and one-year mortality rates. On the other hand, after adding troponin, all risk scores showed similar accuracy, but still acceptable or even poor, especially in the assessment of one-year mortality. In the analysis of the incremental value (Table 4 and Figure 3), we observed that there was an incremental value in all risk models studied, especially in the 30-day mortality.

**Table 4:** – Addition of troponin to risk scores by using the net reclassification index (NRI)

	SR x SR + troponin			RCRI x RCRI + troponin			CVR x CVR + troponin		
			
	NRIe	NRIne	NRI	NRIe	NRIne	NRI	NRIe	NRIne	NRI
30-day mortality	0.48	0.08	0.40	0.48	0.08	0.40	0.48	0.08	0.40
1-year mortality	0.30	0.07	0.23	0.30	0.07	0.23	0.30	0.07	0.23

SR: surgical risk; RCRI: revised cardiac risk index; CVR: cardiovascular risk; NRIe: NRI of events; NRIne: NRI of non-events.

**Figure 3:** – Graphical representation of integrated discrimination improvement for each score in two different outcomes (30-day mortality and 1-year mortality). The area under the curve (AUC) of the IDI plot is a measure of the improvement (red area) in discrimination achieved by the new test over the old test; a larger AUC indicates a greater improvement in discrimination; p < 0.001 in all curves; numeric values are available in supplementary material (Table S2).

## Discussion

In this study, we observed a higher prevalence of myocardial injury in patients at higher risk, including cardiovascular, surgical, and clinical risk. However, the occurrence of MINS in the non-high-risk population is not negligible and causes high mortality in this population. In the long-term follow-up, non-high-risk patients with myocardial injury had a worse prognosis than high-risk ones without myocardial injury. Traditional risk assessment has shown low accuracy in predicting death at 30 days and at one year. On the other hand, the addition of high-sensitivity troponin to the investigation of myocardial injury allowed increasing the accuracy of the prediction of these events, especially in the non-high-risk population.

The occurrence of MINS is known to increase the risk of death in the short and long terms,^5^ but the role of risk assessment in such increase has been little studied. By analyzing the risk factors individually, we know that hypertension, diabetes, and smoking increase the risk of MINS.^9^ However, there is no study evaluating the occurrence of MINS in patients at high cardiovascular risk. In the initial assessment of the patient-related preoperative risk, guidelines recommend the assessment of risk factors and presence of established cardiovascular disease.^3^ For a patient aged ≥ 65 years with risk factors or established cardiovascular disease, it is recommended to perform an electrocardiogram and measure biomarkers (troponin and BNP). However, these data are not used in risk scores.

In our study, the prevalence of high cardiovascular risk was considerable (26.1%), as was the prevalence of hypertension (62.8%) and diabetes (25.7%). High cardiovascular-risk patients had a higher prevalence of MINS (40.1 x 24.8%). In contrast, 30-day mortality rate of high cardiovascular-risk patients without MINS was similar to that of patients without high cardiovascular risk (2.4 x 1.4%). Therefore, we observed that almost a quarter of the population without high cardiovascular risk had MINS, which determined a higher mortality at 30 days (14%) and at one year (29.8%), and demonstrated the need for MINS screening even in patients without high cardiovascular risk. Even when adjusting for severity, these patients had a worse prognosis in a follow-up of almost seven years.

When we evaluated patients undergoing high-risk surgery, we found similar results. Although some risk scores include the intrinsic risk of surgery,^3^ little data is available regarding the occurrence of MINS and its prognostic impact. In our population, the prevalence of high-risk surgery was 14.6%, and these patients had a higher prevalence of MINS (21.3% vs. 13.9%). In the absence of MINS, their 30-day mortality rate was similar to that of patients undergoing lower-risk surgeries (2.5% vs. 1.5%). However, the occurrence of MINS increases the risk of death regardless of the surgery performed, with a 30-day mortality rate of 12.6% in patients undergoing lower-risk surgery. This finding was consistent throughout the study period, indicating that assessing the risk exclusively by the analysis of the inherent risk of surgery is inadequate.

Lastly, we analyzed high clinical-risk patients by using the RCRI, one of the most used pre-operative risk scores in the clinical practice. In this study, only 0.9% of the patients were considered at high risk (RCRI ≥ 3). Even so, these patients had a higher prevalence of MINS (3.0 x 0.7%). In the 30-day mortality analysis, we observed no death among patients with high RCRI score and without MINS. However, the occurrence of MINS was associated with 16.7% of the deaths in this group. In the analysis of long-term mortality, we observed that the groups with high RCRI (with and without MINS) had a worse prognosis in the follow-up. This finding can be justified by the small sample size of this group (64 patients), in which the occurrence of an event was exacerbated in relation to the other group.

The RCRI, despite being widely used, is not a tool with good accuracy in detecting cardiovascular events, especially all-cause death.^10,11^ The accuracy detected in our study (c-statistics = 0.625 for death at 30 days) is in line with that found in the literature.^3^ However, the addition of post-operative high-sensitivity troponin was able to increase its predictive ability. Vasireddi et al.^12^ demonstrated that patients classified as low risk through the RCRI score showed higher mortality rates when they had myocardial injury,^12^ a finding corroborated by this study. Because low-risk patients are often neglected for protective measures in the pre-operative preparation and, therefore, they could be more exposed to the risk of myocardial injury. This finding was consistent with other risk assessments, such as that of the inherent risk of surgery and cardiovascular risk. Despite the increase in predictive capacity with the addition of high-sensitivity troponin to risk stratification, the accuracy was only considered acceptable (c-statistics between 0.7 and 0.8).^13,14^ Thus, new risk assessment scores including high-sensitivity troponin are still needed.

In the analysis using the NRI, we observed a higher reclassification rate in patients who had myocardial injury, especially regarding the 30-day mortality. This finding corroborates the incremental power of high-sensitivity troponin in the risk reclassification of patients undergoing non-cardiac surgeries. Our findings were further supported by the results of the IDI test. The IDI is a widely used tool for evaluating the ability of a marker to predict binary outcomes. It has been suggested that the IDI is more sensitive than other metrics in identifying useful predictive markers. In our study, high-sensitivity troponin emerged as a powerful predictor of mortality in patients undergoing non-cardiac surgery. This was demonstrated using three distinct statistical methods, adding robustness to our results.

The addition of high-sensitivity troponin to the clinical practice enabled the detection of minor degrees of myocardial injury. In a follow-up, the VISION study demonstrated that elevations of high-sensitivity troponin above 5ng/L during the post-operative period increased the 30-day mortality of non-cardiac patients.^15^ In our study, we demonstrated that low-risk patients are also vulnerable to myocardial injury. On the other hand, the population studied had a potential risk of severe disease in view of their ICU stay longer than one night. Thus, this population deserves routine screening with high-sensitivity troponin dosage in the post-operative period, regardless of the risk, a finding also corroborated by our study.

The present study has some limitations. Despite being a retrospective analysis study, data were prospectively collected from the local database. Different troponin kits were used during this study, making it difficult to standardize the data as a continuous variable. In any case, the recommendation of the American Heart Association is, regardless of the kit used, to use the 99th percentile to characterize patients with MINS. In addition to these limitations, this is a single-center study. Furthermore, when we analyze long-term outcomes, other factors may directly influence the risk of death that cannot be controlled in a retrospective study, adding a high risk of bias. Finally, the selection of patients admitted to ICU demonstrates a potentially higher-risk population and, therefore, our results cannot be extrapolated to other populations.

Despite its limitations, our study is among the few that have assessed the prognosis of patients undergoing non-cardiac surgeries across a broad spectrum of risk. We employed three different risk classifications and demonstrated that even patients considered to be at low risk may be exposed to higher mortality. Our findings highlight the need for more widespread use of high-sensitivity troponin measurements in identifying patients at greater risk. While current scores and risk assessments fail to identify these patients, the addition of high-sensitivity troponin to standard stratification methods was shown to improve the predictive capacity for 30-day and one-year mortality.

## Conclusions

Patients at high risk based on cardiovascular risk, intrinsic risk of surgery or RCRI score had a higher prevalence of myocardial injury when undergoing non-cardiac surgery. Usual risk stratification showed low accuracy in predicting all-cause death in the short and long terms; the addition of high-sensitivity troponin to risk assessment increased the predictive ability, but it is still insufficient for a good prediction of events. New scores using biomarkers should be developed.
